# Effective elimination of Moiré fringe at the nanoprecipitate/matrix interface

**DOI:** 10.1186/s42649-025-00121-7

**Published:** 2026-01-07

**Authors:** Yoon-Uk Heo, Dongwon Lee, T. T. T. Trang, Changwan Hong

**Affiliations:** 1 Graduate Institute of Ferrous and Echo Materials Technology, Pohang University of Science and Technology, Cheongam-Ro 77, Hyoja-Dong, Pohang 37-673 Republic of Korea; 2 MX Division, Samsung Electronics, Samsungro 129, Suwon-Si, Gyeonggi-Do 16677 Republic of Korea; 3 Daegu Mechatronics & Materials Institute, 11 Seongseogongdan-Ro, Daegu, 42714 Republic of Korea

**Keywords:** Moiré fringe, Nano precipitate, TEM, GPA, MC carbide

## Abstract

The effective method for eliminating the Moiré fringes in the MC nanoprecipitates in the austenite matrix was studied. Considering the dynamic diffraction between the matrix and precipitate, the spots originating from the Moiré pattern were selected in the fast Fourier transform (FFT) pattern. The Moiré fringe image was extracted by performing inverse FFT(IFFT) of the selected spots. Subtracting the Moiré fringe contribution from the original image intensity reveals the veiled lattice fringes of MC carbide in the austenite matrix. The image was compared and discussed with the IFFT image of the direct selection of the matrix + MC FFT patterns. The suggested method was applied to strain measurement in the MC carbide-containing area using the geometrical phase analysis. Compared with the result obtained from the raw image containing Moiré fringes, the applied method shows artifact-free strain distribution in the MC carbide. The process was applied to the inclined twin interface in BCC steel. The lattice fringe in the overlapping area shows a more detailed lattice feature. This novel study sheds light on the veiled crystal structure and strain distribution under various conditions where more than two lattice fringes overlap.

## Introduction

The current environmental issues demand higher-strength and higher-toughness alloys for applications in extreme conditions. Multiple strengthening mechanisms, including grain boundary strengthening (Heo et al. 2018), solid solution strengthening (Leslie 1972), precipitation hardening (Ardell 1985), and strain hardening (Fang & Dahl 1995), are employed to design the microstructure and achieve the required mechanical properties. Among various strengthening mechanisms, precipitation hardening enhances both yield and ultimate tensile strengths without a significant loss of ductility (Altuna et al. 2012). Precipitation of nano-sized particles not only introduces a hetero-structured area (precipitate) but also generates a strain field in the surrounding matrix (Martin 1998). The formed nano-sized precipitates act as an effective barrier to dislocation movement, thereby increasing strength (Gladman 2013). Since each precipitate has a different bulk modulus (Frantsevich & Lyashenko 1966) and coherency with the matrix, the strengthening effect of the precipitate varies depending on the type of precipitate and the matrix. Therefore, identifying the hardening phase and strain field in the matrix is a critical step in designing high-strength alloys by introducing effective precipitates into the matrix.

The identification of the nano-sized precipitate is achieved by analyzing the electron diffraction pattern (EDP) or fast Fourier transform (FFT) pattern of a high-resolution (HR) image in the precipitate-containing area using transmission electron microscopy (TEM) (Heo et al. 2009; Hong et al. 2016). However, the size of the precipitate (< a few tens of nm) is generally smaller than the thickness of the TEM specimen (*t* < 100 nm). Dynamic diffraction, or Moiré fringes, arising from the interaction between the matrix and nano precipitate, makes it challenging to identify the precipitate’s crystal structure (Casillas et al. 2020; Heo et al. 2013; Trang et al. 2023). Moiré fringe also affects the evaluation of the strain field in the precipitate due to the contrast interference in the HR image (Cautaerts et al. 2019). On the contrary, studies showed that the application of Moiré fringes for deformation analysis has improved HRTEM's capability for quantitative defect characterization. The subset geometric phase analysis (GPA) method improved the extraction of local phase information and enabled accurate mapping of highly non-uniform strain fields (Zhang et al. 2016). Nano-Moiré techniques were further developed to visualize and evaluate deformation within weakly imaged or even invisible lattice regions near grain interfaces, allowing access to structural information beyond conventional lattice imaging (Zhang et al. 2017). Digital phase-shifting Moiré methods introduced computational phase modulation, enabling precise identification of dislocations, interfaces, and real strain distributions (Zhu et al. 2019). More recently, hybrid approaches have been tried to obtain the integrated lattice image reconstruction with phase-based deformation analysis, substantially reducing noise sensitivity and improving the reliability of strain quantification in complex microstructural environments (Zhang et al. 2022). Even though these constructive applications of Moiré fringes existed, the direct understanding of the veiled microstructure is still confusing due to the random or complicated formation of the fringes, depending on the combination of two lattices and their orientation relationship. To clarify the precipitate's phase and strain fields, eliminating the Moiré fringes in the raw image is necessary.

In this study, we studied the novel pathway of eliminating Moiré fringe to reveal the original HR image of MC precipitate in austenite (γ) matrix. Based on the understanding of the principle of Moiré fringe formation, the contrast of the Moiré fringe is extracted from the raw image. The Moiré fringe-free contrast was obtained by subtracting the Moiré fringe contrast from the raw image. The obtained image was used to evaluate the strain field through geometrical phase analysis (GPA). The method was also applied to reveal the original structure in the inclined twin boundary of 12Mn steel. The Moiré fringe-eliminated image clarified the overlapped lattice fringes of the matrix and twin at the inclined interface. This novel study sheds light on understanding the veiled crystal structure and strain distribution under various conditions in which more than two lattice fringes overlap.

## Materials and methods

TP347H austenitic stainless and 12Mn-containing steels (12Mn) were prepared by vacuum induction melting. The chemical compositions of both steels are listed in Table 1. The conventional hot-rolling process was applied to produce a thick plate. Then, TP347H austenitic stainless steel was machined into a tensile specimen with dimensions of 100 × 10 × 10 mm. The rupture test was conducted at 750 °C for 571 h under an applied stress of 80 MPa. On the other hand, 12Mn steel was solution treated at 900 °C and water-quenched. Both specimens were cut into a thin plate and mechanically polished to a thickness of 100 μm. 3 mmϕ disc was punched out from the thin plate. Electrochemical polishing was performed in a solution of 10% perchloric acid and 90% acetic acid at 35 V at room temperature. To eliminate the chemical etching effect, the thin foil specimen was further milled with Ar + ions at 4.5 keV for 1 h using a precision ion polishing system (Gatan 691 PIPS). Precipitate in the matrix was investigated using a field-emission TEM (JEOL, JEM-2100F, Tokyo) equipped with an energy-dispersive spectrometer (EDS, Aztech, Oxford instrument) at 200 kV.

**Table 1:** Chemical compositions of the alloys (wt. %)

Alloy	**Mn**	**S**	**C**	**Cr**	**Ni**	**Nb**	**P**	**Si**	**N**	**Fe**
TP347H	1.51	0.0093	0.071	18.12	10.05	0.48	0.030	0.609	0.03	Bal
12Mn	11.71	< 0.001	0.055	-	-	-	< 0.001	-	< 0.02	Bal

HR images were recoded using the Digital Micrograph (DM) software (Ver. 3.40, Gatan). Image post-processing was conducted in the DM. The obtained HR images were processed by following the sequences: i) FFT of the HR image, ii) mask with a circular-shaped mask in FFT pattern, iii) Inverse FFT of the masked spots. GPA analysis was performed on the HR images using the GPA plug-in program ver. 4.12.1 (HREM Research Inc.). The corresponding spots were selected by applying the circular mask. Then, the image was Bragg-filtered, and the Fourier-transformed images were generated. Finally, by choosing a reference area, fringe deformation mapping was obtained.

## Results and discussion

### Principle and elimination method of the Moiré fringe formed on the MC carbide region in the γ-matrix

**Fig. 1:**
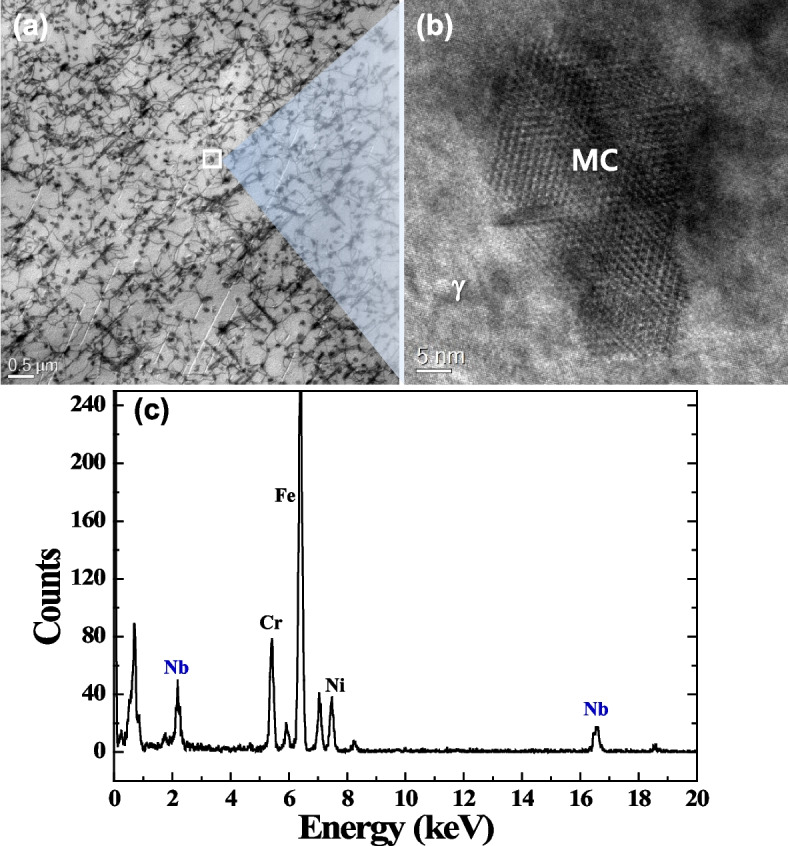
TEM images of fine precipitates in the γ-matrix: **a** bright field (BF)-scanning TEM(STEM) image, **b** HR-TEM images of an MC precipitate, and **c** its EDS spectrum

Figure 1a shows the BF-STEM image of fine precipitates in the γ-matrix. Nano-sized precipitates interact with dislocations. HR-TEM image of a precipitate in Fig. 1b shows a well-developed Moiré fringe that reflects the overlapping of two lattice fringes (γ-matrix and MC). The EDS spectrum in Fig. 1c proves that these nanoprecipitates are an NbC-typed MC carbide.

**Fig. 2:**
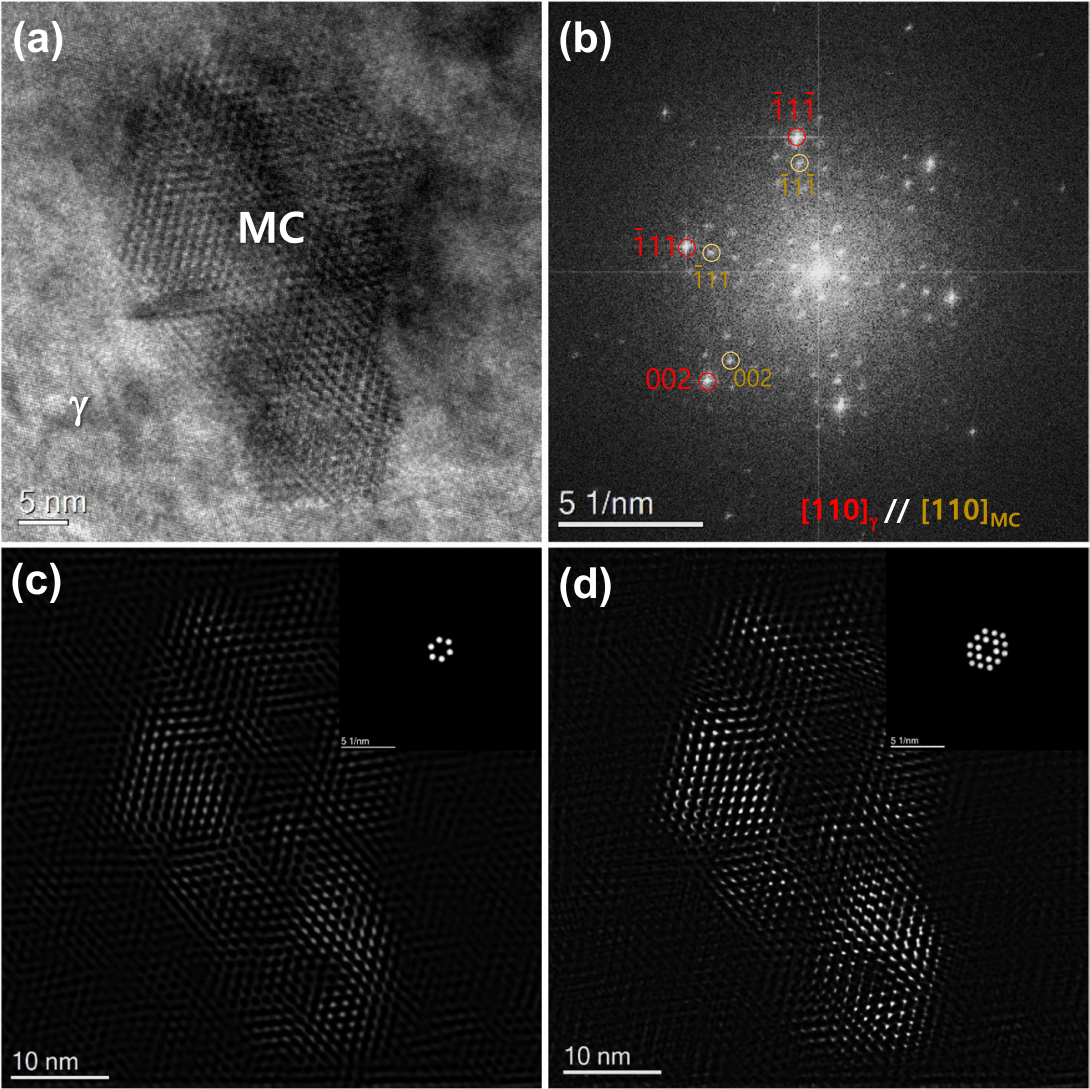
Analysis of Moiré fringe on the MC nanoprecipitate in the γ-matrix; **a** raw image, **b** FFT pattern, and **c**, **d** IFFT images including the first-order and first + second-order Moiré spots

The Moiré fringe was further analyzed using the FFT pattern and inverse FFT(IFFT) image, as shown in Figs. 2a to d. The FFT pattern in Fig. 2b reveals the cube-cube orientation relationship (OR) between Nb-rich MC carbide and the γ-matrix. There are many spots besides those from the MC carbide and γ-matrix. The unindexed spots in Fig. 2b exhibit a well-defined spacing, inherited from the periodicity in the HR image. The IFFT images of the first order and first + second order spots clarify where the unindexed spots are related to Moiré fringes (Figs. 2c and d). The IFFT image contrast is dominant at the MC, where the MC and γ-matrix lattice fringes overlap.

**Fig. 3:**
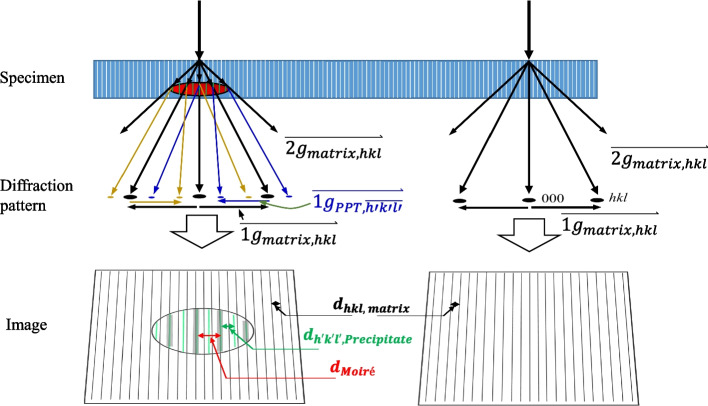
Schematic description of Moiré fringe formation in the nanoprecipitate-bearing alloy

The formation of the Moiré fringe is known to originate from the overlapping of two different lattice fringes (Latychevskaia et al. 2019; Williams & Carter 2009). Similarly, the Moiré pattern is also understood as the result of dynamic diffraction (double diffraction) in two different crystals (Bassett et al., 1997; Hashimoto et al. 1961). Figure 3 shows the schematic description of Moiré fringe formation in the electron diffraction pattern and the HR image. Dynamic diffraction from the matrix and precipitate forms the Moiré spots in the electron diffraction pattern. The sum of $\overrightarrow{{1g}_{matrix, hkl}}$ and $\overrightarrow{1g_{PPT,\overline{h'k'l'}}}$ vectors makes the Moiré spot near the 000 spot. Similary, $\overrightarrow{{2g}_{matrix, hkl}}$ + $\overrightarrow{1g_{PPT,\overline{h'k'l'}}}$ forms Moiré spot near the hkl_matrix_ spot. Moiré fringes are shown in HR image of the precipitate (Fig. 3), where the dynamic diffraction occurs. Besides the lattice fringes of the matrix and precipitate, a thick Moiré fringe is frequently observed in the overlapped area (Fig. 3).

**Fig. 4:**
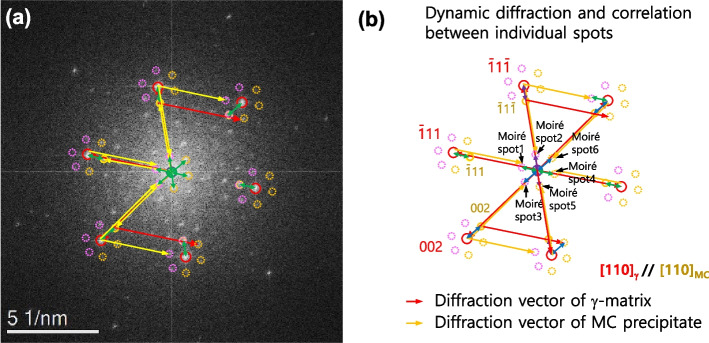
Understanding the formation principle of the Moiré spots: **a** FFT pattern of Fig. 2a and **b** its indexing of Moiré spots based on the dynamic diffractions

Figure 4a shows the formation of Moiré spots in the FFT pattern of Fig. 2a. The sum of diffraction vectors in the γ-matrix (red arrow) and the MC precipitate (yellow arrow) forms the Moiré spots near the 000 and *hkl*_*matrix*_ spots. The detailed relation between the diffraction and Moiré spots is described in Fig. 4b. Since the periodic spacing of the Moiré fringe corresponds to the Moiré spots of 1 to 6 in Fig. 4b, the second-order Moiré spots can also be formed in Fig. 4a.

**Fig. 5:**
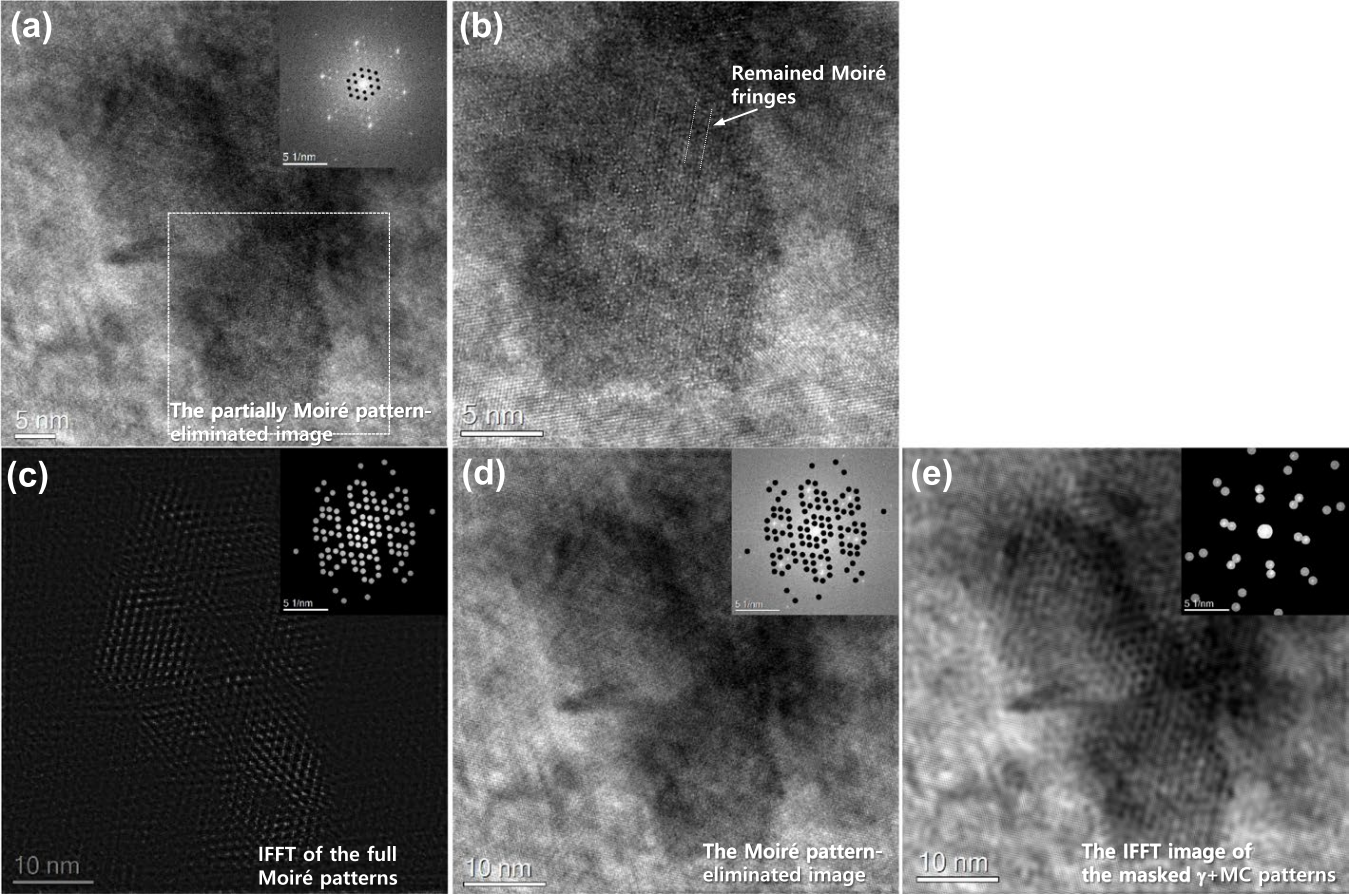
Construction of the Moiré fringe-eliminated HR image: **a** the image subtracted Fig. 2d from Fig. 2a, **b** the magnified image of the square region in **a**, **c** the complete Moiré fringe contrast, **d** the image subtracted Fig. 3c from Fig. 2a, and **e** IFFT image of the masked γ + MC patterns. Note that the corresponding FFT patterns are inserted with the insets

Based on the understanding of the formation principle of the Moiré spots, the Moiré fringe-eliminated image was extracted in Fig. 5. Figure 5a is constructed by subtracting Fig. 2d from Fig. 2a. The Moiré spots near the transmitted spot (000) were removed from the raw image. It looks like the repeated Moiré fringes have been eliminated. However, the magnified image in Fig. 5b shows that the MC precipitate still exhibits a residual Moiré fringe. All the Moiré spots, including the spots formed near the *hkl*_*matrix*_ spots, were selected and used for the IFFT in Fig. 5c. The constructed complete Moiré contrast was subtracted from the raw image. The Moiré fringe-eliminated image is shown in Fig. 5d. The remaining Moiré contrast is entirely removed in the image. Moreover, the Moiré fringe-subtracted image in Fig. 5d includes the contribution of inelastic scattering (diffuse intensity of the background in the FFT pattern). This makes the image close to the raw image, even though the process includes masking and IFFT of specific spots. For reference, the IFFT image of the masked γ + MC patterns was displayed in Fig. 5e. Comparing Fig. 5e with Fig. 5d, it is demonstrated that the direct selection of γ + MC patterns for the IFFT cannot ideally eliminate the Moiré fringes.

### Application of the Moiré fringe elimination to reveal the intrinsic structure of materials

**Fig. 6:**
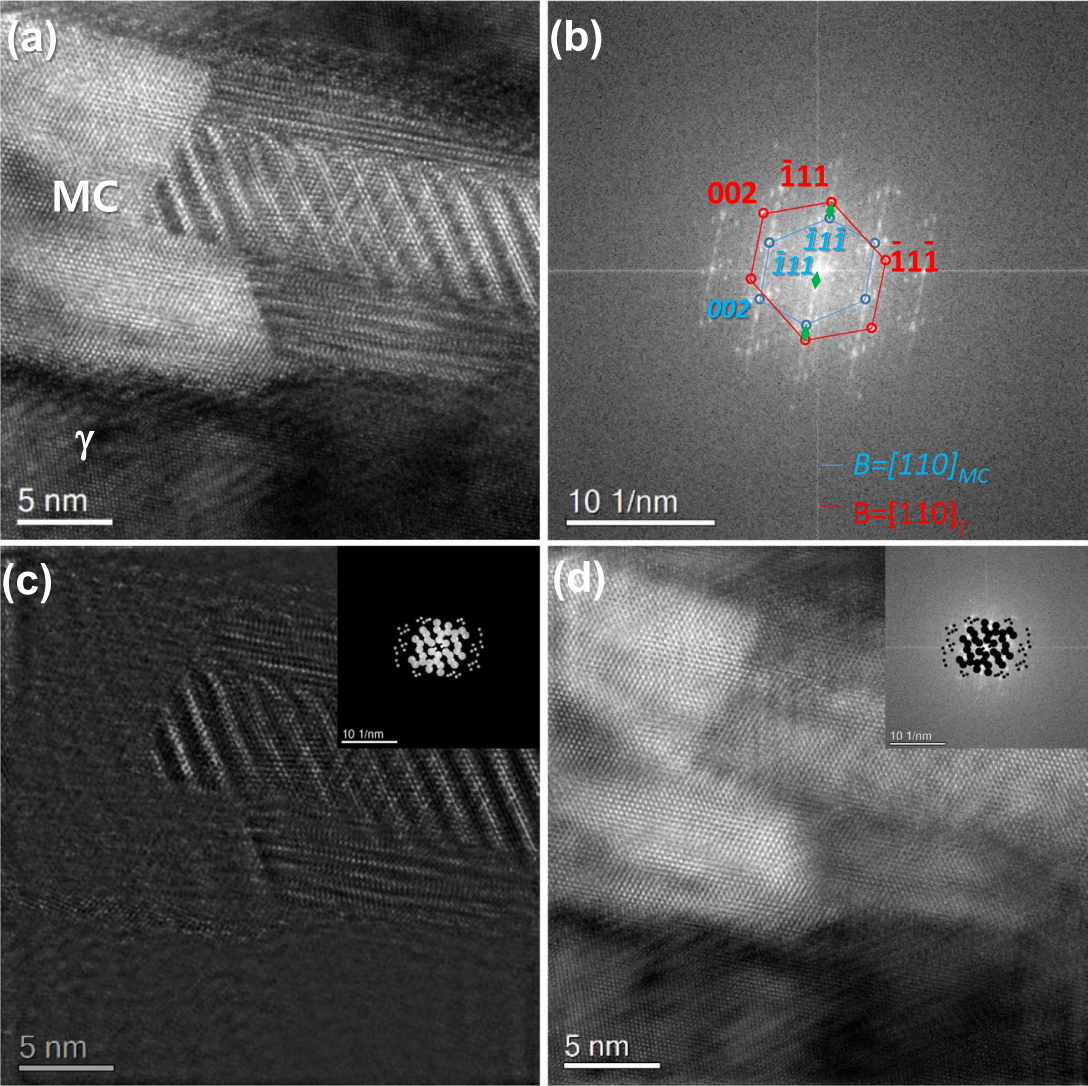
Analysis of MC carbide precipitate: **a** HR image and **b** its FFT pattern of partially overlapped MC carbide with the γ-matrix, **c** Moiré fringe contrast, and **d** the Moiré fringe-eliminated image with its FFT pattern

**Fig. 7:**
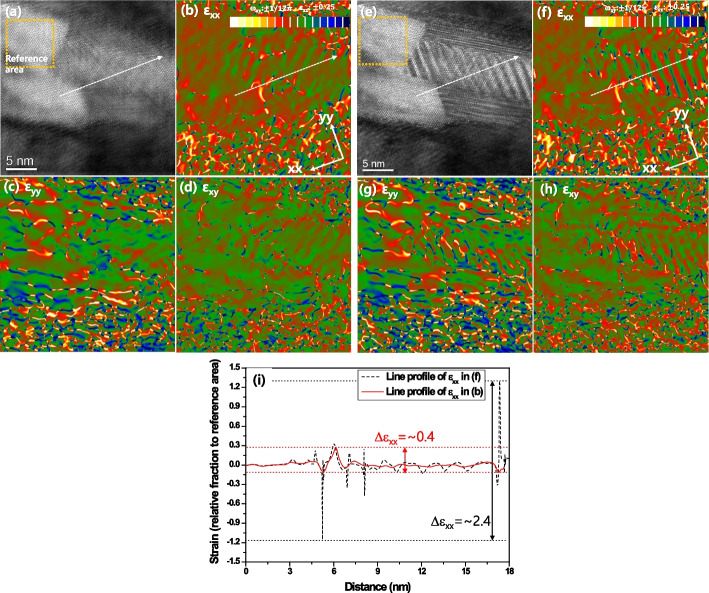
Comparison of GPA analyses in Moiré fringe-containing and -removed images: **a** Moiré fringe-removed HR image and **b**, **c**, **d** its strain maps, **e** Moiré fringe-containing HR image and **f**, **g**, **h** its strain maps, and **i** line profiles of dash lines in **b** and **f**

The formation of Moiré fringes depends on the combination of the matrix and precipitate phases. Even though the same combination of phases, the formation positions of Moiré spots can also change with the orientation relationship between them. Figure 6a shows an HR image of the MC carbide that is partially covered with the γ-matrix. The FFT pattern of Fig. 6a clarifies that the MC precipitate holds the twin-like OR with the γ-matrix (Fig. 6b). The Moiré spots are different from Fig. 2b. Figure 2b shows the formed Moiré spots under the cube-cube orientation relationship between the γ-matrix and MC phase. However, the Moiré spots changed in Fig. 6b because they have a twin-like orientation relationship. Since the combinations of phase cases and their orientation relationships are countless, the researcher should carefully identify Moiré spots using the crystallographic data of both phases. The Moiré spots are carefully selected and used for the construction of the Moiré fringe contrast in Fig. 6c. The Moiré contrast is removed from the raw image by subtracting Fig. 6c from Fig. 6a. The obtained image in Fig. 6d shows clean lattice fringes without the Moiré fringes. The results of GPA analyses in both raw and Moiré fringe-subtracted images were further compared in Fig. 7. As shown in Fig. 7f to h, a substantial contribution of Moiré fringe is observed in the strain maps. Modulated Moiré contrast effect transfer in the strain maps. However, the Moiré fringe-originated artifact is removed in the strain maps when the Moiré fringe is eliminated in the HR image (Figs. 7a to d). The line profiles of strain ε_xx_ reveal these changes, as shown in Fig. 7i. The fluctuation of strain reaches about 2.4 in the MC particle when the raw image was evaluated. However, the deviation of the strain at the same positions was reduced to about 0.4 with the elimination of the Moiré fringe effect. Therefore, eliminating the Moiré fringe can reveal the intrinsic structure and strain distribution in the overlapped area of the multi-lattices, including the inclined boundary.

**Fig. 8:**
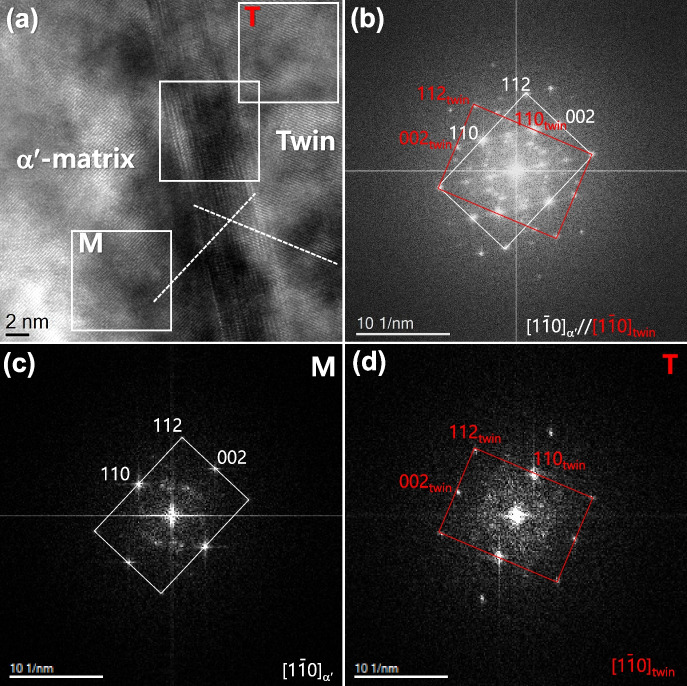
Moiré fringe formed at the inclined twin boundary in BCC 12Mn steel: **a** HR image and FFT patterns of **b** twin boundary, **c** matrix, and **d** twin areas

**Fig. 9:**
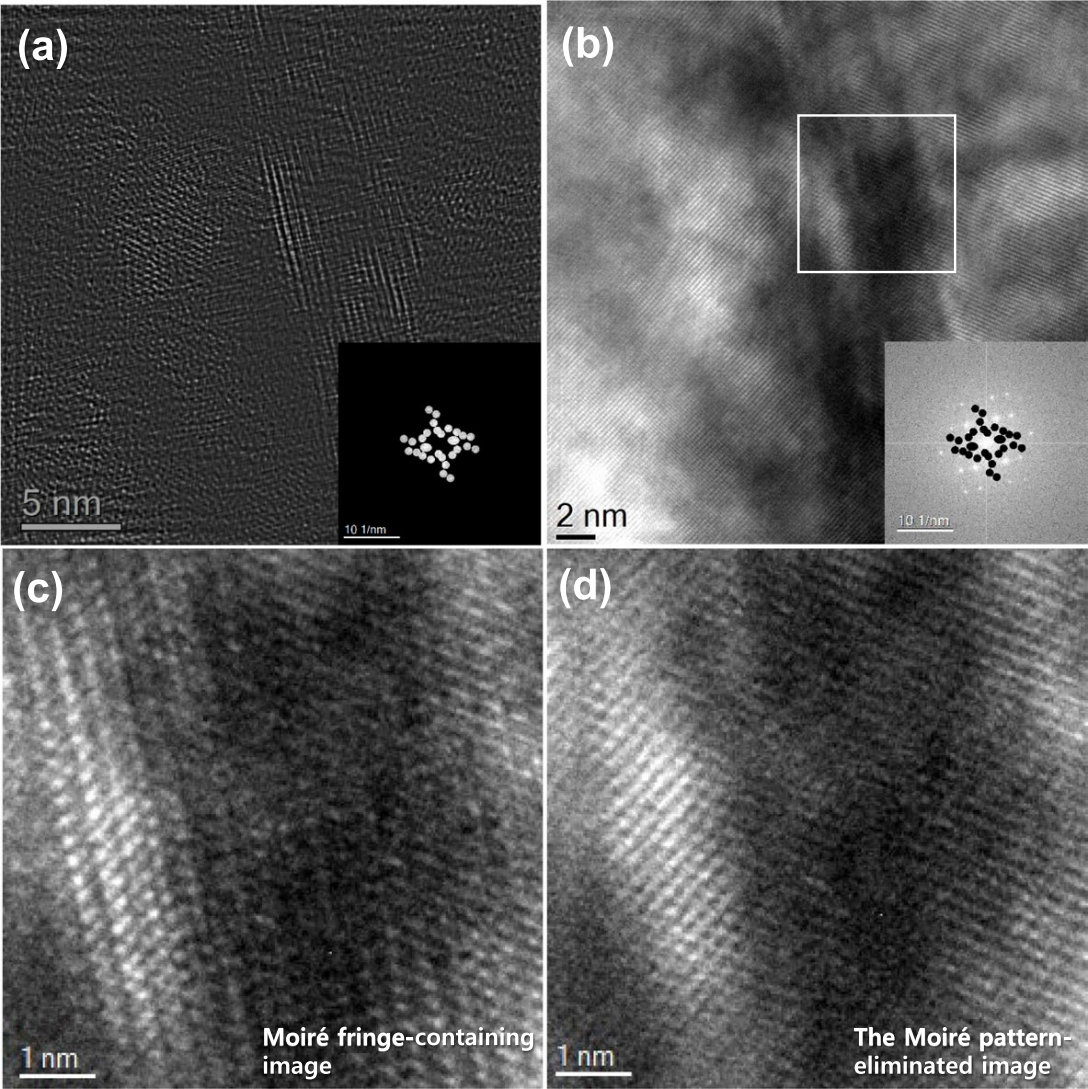
Application of the Moiré fringe elimination on the inclined twin boundary in the martensitic 12Mn steel: **a** Moiré fringe contrast, **b** Moiré fringe-eliminated image, and **c**, **d** the magnified images of the selected region in Figs. 8a and Fig. 9b, respectively

Figure 8a shows an HR image that forms at the inclined twin boundary in 12Mn steel. FFT patterns of martensite(α′) matrix, twin, and twin boundary areas clarify the twin relationship (Figs. 8b to d). The HR image of the twin boundary was misinterpreted as the ω phase because the lattice fringes and diffraction pattern were similar to those of the ω phase (Liu et al. 2015; Ping et al. 2018). However, multi-directional investigation of the twin boundary clarified the ledge-type of twin boundary (Trang et al. 2023). Figure 9a shows the IFFT image of the Moiré spots in the FFT pattern of Fig. 8a. The Moiré fringe-eliminated image was obtained in Fig. 9b by subtracting Fig. 9a from Fig. 8a. Magnified images of the selected regions in Fig. 8a and Fig. 9b were compared in Figs. 9c and d. The repeated strong lattice fringe (Fig. 9c), which misleads to the ω phase, in the raw image, was clearly eliminated in Fig. 9d. The overlapping of both matrix and twin lattice fringes is revealed in the Moiré fringe-eliminated image (Fig. 9d).

**Fig. 10:**
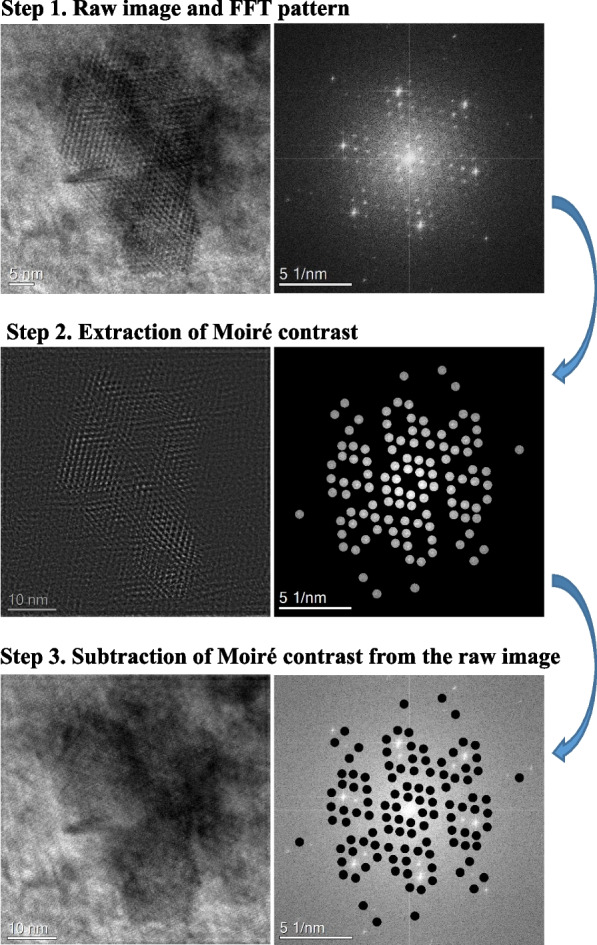
Procedure of the Moiré-fringe elimination from the raw image

The procedure of the Moiré fringe-elimination is displayed in Fig. 10. The FFT pattern is first obtained from the raw image. In the FFT pattern, we identify the matrix and precipitate spots using their crystallographic information. All the other spots are considered as Moiré spots. The Moiré spots are masked and used to extract Moiré contrast. The IFFT of the masked spots forms the Moiré contrast. The Moiré fringe-eliminated image is obtained by subtraction of the Moiré contrast from the raw image.

## Conclusion

The novel pathway to achieving Moiré-fringe-free contrast was studied using the MC nano precipitate in TP347H austenitic stainless steel. The following are the conclusions obtained from this study;

1. The principles of Moiré fringe and Moiré spot formations were understood by dynamic(double) diffraction of the incident beam with both matrix and precipitate lattices.
2. Moiré spots form near the 000 and *hkl*_*matrix*_ spots by dynamic diffraction of the first and *nth* order matrix diffraction with precipitate diffraction.
3. The Moiré contrast can be extracted by the IFFT of all the selected Moiré spots.
4. Simple subtraction of Moiré contrast from the raw image forms a Moiré fringe-eliminated HR image. The obtained image differs from the direct IFFT image of the matrix and precipitate spots, in which the Moiré fringe persists in the overlapped region.
5. The Moiré fringe-eliminated image shows an artifact-free strain map in the GPA analysis. The suggested method also reveals the veiled lattice structures in the inclined twin boundary in martensitic 12Mn steel.

## Data Availability

The datasets used and/or analyzed during the current study are available from the corresponding author on reasonable request.

## Abbreviations

FFT: Fast Fourier transform
IFFT: Inverse fast Fourier transform
EDP: Electron diffraction pattern
HR-TEM: High-resolution transmission electron microscopy
GPA: Geometrical phase analysis
